# A comparison between the abdominal and femoral adipose tissue proteome of overweight and obese women

**DOI:** 10.1038/s41598-019-40992-x

**Published:** 2019-03-12

**Authors:** M. A. A. Vogel, P. Wang, F. G. Bouwman, N. Hoebers, E. E. Blaak, J. Renes, E. C. Mariman, G. H. Goossens

**Affiliations:** 1 Department of Human Biology, NUTRIM School of Nutrition and Translational Research in Metabolism, Maastricht University Medical Center+, Maastricht, The Netherlands; 2 Department of Clinical Genetics, Maastricht University Medical Center+, Maastricht, The Netherlands

## Abstract

Body fat distribution is an important determinant of cardiometabolic health. Lower-body adipose tissue (AT) has protective characteristics as compared to upper-body fat, but the underlying depot-differences remain to be elucidated. Here, we compared the proteome and morphology of abdominal and femoral AT. Paired biopsies from abdominal and femoral subcutaneous AT were taken from eight overweight/obese (BMI ≥ 28 kg/m^2^) women with impaired glucose metabolism after an overnight fast. Proteins were isolated and quantified using liquid chromatography-mass spectrometry, and protein expression in abdominal and femoral subcutaneous AT was compared. Moreover, correlations between fat cell size and the proteome of both AT depots were determined. In total, 651 proteins were identified, of which 22 proteins tended to be differentially expressed between abdominal and femoral AT after removal of blood protein signals (p < 0.05). Proteins involved in cell structure organization and energy metabolism were differently expressed between AT depots. Fat cell size, which was higher in femoral AT, was significantly correlated with ADH1B, POSTN and LCP1. These findings suggest that there are only slight differences in protein expression between abdominal and femoral subcutaneous AT. It remains to be determined whether these differences, as well as differences in protein activity, contribute to functional and/or morphological differences between these fat depots.

## Introduction

Obesity is related to cardiometabolic disorders that contribute to increased morbidity and mortality^1,2^. Being a highly active metabolic and endocrine organ^3^, adipose tissue (AT) is involved in the regulation of many physiologic processes, like immune responses, energy balance, blood pressure regulation, and glucose homeostasis^4^. The expansion and remodeling of AT during excessive weight gain renders the tissue dysfunctional^5^. AT dysfunction in obesity is strongly linked to metabolic dysregulation and increased risk of cardiometabolic diseases^5,6^.

In addition to total AT mass, the location where lipids are stored seems an important determinant of the cardiometabolic consequences^7,8^. Contrary to central obesity, accumulation of lower-body fat appears protective against metabolic derangements and hypertension^9^, and is associated with a reduced incidence of type 2 diabetes mellitus and cardiovascular disease when adiposity is comparable^10,11^. However, the underlying mechanisms for the differences in disease risk associated with a certain body fat distribution remain elusive. We have recently demonstrated that abdominal subcutaneous adipose tissue is characterized by smaller adipocytes and a distinct pattern of gene expression compared to femoral adipose tissue in overweight/obese women, which may contribute to functional differences between these fat depots^12^.

Omics methodology provides excellent opportunities to investigate putative differences between AT depots. Microarray analysis of gluteofemoral (GFAT) and abdominal AT revealed that expression of energy-generating metabolic genes was inversely, and of inflammatory genes was positively associated with obesity^13^. Interestingly, for GFAT, the association between AT inflammation and BMI was weaker as compared to abdominal AT, which was confirmed by a lower secretion of interleukin-6 from lower-body AT. Moreover, markers of macrophage infiltration were not enriched in GFAT but increased in abdominal AT with obesity^13^.

To investigate AT depot-differences at a more functional level, proteomics analysis may be highly valuable. It has previously been shown that proteins related to metabolic processes such as glucose and lipid metabolism, lipid transport, protein synthesis, protein folding, response to stress and inflammation were differentially expressed in abdominal subcutaneous as compared to omental AT in humans^14^. Furthermore, proteome differences in either subcutaneous or visceral AT in relation to BMI or metabolic health have been investigated in humans^15–18^. In this respect, it has previously been found that several proteins related to AT remodeling, including several keratin and annexin proteins, and proteins related to oxidative stress were more abundant in the abdominal AT of obese and overweight as compared to lean individuals, both in men and women^16^. Although structural and functional differences between visceral and subcutaneous adipose tissue may be more pronounced than differences between different subcutaneous AT depots, a direct comparison of the proteome of upper- and lower-body subcutaneous human AT has not been performed yet.

In the present study, we compared for the first time, to our knowledge, the proteome of abdominal and femoral subcutaneous AT in overweight and obese women with impaired glucose metabolism using untargeted quantitative liquid chromatography-mass spectrometry to obtain insights in the physiological differences between these subcutaneous AT depots in humans.

## Materials and Methods

### Subjects

Eight overweight and obese (BMI ≥ 28 kg/m^2^) women with an impaired fasting glucose (IFG: fasting plasma glucose 5.6–7.0 mmol/l) or impaired glucose tolerance (IGT: 2 h plasma glucose 7.8–11.1 mmol/l) participated in the present study. Exclusion criteria were smoking, cardiovascular disease, type 2 diabetes mellitus, liver or kidney disease, use of medication known to affect body weight and glucose metabolism, marked alcohol consumption (>14 alcoholic units/wk). Furthermore, subjects had to be weight stable (weight change <3.0 kg) for at least three months prior to the start of the study. Subjects were asked to refrain from strenuous physical activity for at least two days before biopsies were collected and measurements were performed.

The study was performed according to the declaration of Helsinki and was approved by the Medical-Ethical Committee of Maastricht University. All subjects gave their written informed consent before participation in the study.

### Anthropometric measurements

Body weight was measured to the nearest 0.1 kg (Seca, Hamburg, Germany). Height was measured using a wall-mounted stadiometer (model 220; Seca, Hamburg, Germany). Waist (top of the iliac crest) and hip (widest portion of the buttocks) circumferences were measured. Body composition and body fat distribution were determined by DEXA (Hologic QDR 4500-A, Waltham MA, USA).

### Adipose tissue biopsies

Abdominal and femoral subcutaneous AT biopsies (~1 g) were collected using needle aspiration under local anaesthesia (2% lidocaine), 6–8 cm lateral from the umbilicus and from the lateral site of the upper leg, respectively, after an overnight fast. Biopsies were immediately rinsed with sterile saline and visible blood vessels were removed with sterile tweezers. The tissue was snap-frozen in liquid nitrogen and stored at −80 °C until analysis.

### Adipocyte morphology

Histological sections were cut from paraffin-embedded AT, and stained with haematoxylin and eosin. Fat cell size was measured using digital images that were captured and analyzed using a Leica DFC320 digital camera (Leica, Rijswijk, Netherlands) and software (Leica QWin V3, Cambridge, United Kingdom), as described before^12^.

### Protein isolation and preparation for LC-MS

Frozen AT (~100 mg) was ground in a mortar with liquid nitrogen. Per microgram of grounded powder, 2 μl of 50 mM ammonium bicarbonate with 5 M urea was added to dissolve the powder. The solution was freeze-thawed in liquid nitrogen 3 times after which it was vortexed for 5 min. The homogenate was centrifuged at 20,000 g for 30 min at 10 °C. The supernatant was carefully collected and protein concentrations were determined with a Bradford-based protein assay (Bio-Rad, Veenendaal, the Netherlands). A control sample was prepared from a pool of 10 μl of each sample.

Samples were digested with Trypsin (Promega) and peptides from 100 µg protein were labelled with TMT isobaric mass tagging labelling reagent (10-plex; Thermo Scientific, West Palm Beach, FL, USA) according to the manufacturer’s protocol. Briefly, 100 μg of protein was used for each sample. The TMT labeling reagents were dissolved in 41 μl acetonitrile per vial. The reduced and alkylated samples and control were added to the TMT reagent vials. The reaction was incubated for 1 h at room temperature and quenched for 15 min by adding 8 μl of 5% hydroxylamine, as described previously^19^. Equal amounts of the 16 samples from 8 subjects (biological replicates) were combined and analyzed by LC-MS in two injections; each injection composed of a mixture of 8 samples from 4 subjects and the control.

### Protein quantification using LC-MS

A nanoflow HPLC instrument (Dionex ultimate 3000) was coupled on-line to a Q Exactive HF (Thermo Scientific) with a nano-electrospray Flex ion source (Proxeon). One μg of TMT labeled peptide mixture was loaded onto a C18-reversed phase column (Thermo Scientific, Acclaim PepMap C18 column, 75-μm inner diameter x 15 cm, 2-μm particle size). The peptides were separated with a 150 min linear gradient of 4–50% in buffer A (100% water with 0.1% TFA) with buffer B (80% acetonitrile and 0.08% formic acid) at a flow rate of 300 nL/min.

MS data was acquired using a data-dependent top-10 method, dynamically choosing the most abundant precursor ions from the survey scan (280–1400 m/z) in positive mode. Survey scans were acquired at a resolution of 60,000 and a maximum injection time of 120 ms. Dynamic exclusion duration was 30 s. Isolation of precursors was performed with a 1.8 m/z window and a maximum injection time of 200 ms. Resolution for HCD spectra was set to 30,000 and the Normalized Collision Energy was 30 eV. The under-fill ratio was defined as 1.0%. The instrument was run with peptide recognition mode enabled, but exclusion of singly charged and charge states of more than five.

### Database search

The MS data were searched using Proteome Discoverer 2.1 Sequest HT search engine (Thermo Scientific), against the UniProt human database. The false discovery rate (FDR) was set to 0.01 for proteins and peptides, which had to have a minimum length of 6 amino acids. The precursor mass tolerance was set at 10 ppm and the fragment tolerance at 0.02 Da. One miss-cleavage was tolerated, oxidation of methionine was set as a dynamic modification and carbamidomethylation of cysteines, TMT reagent adducts (+229.162932 Da) on lysine and peptide amino termini were set as fixed modifications.

### Data quantification and normalization

The MS-acquired data were first analyzed with Thermo Scientific Proteome Discoverer software version 2.1. Relative quantitation of peptides from mixed samples was extracted by comparing the signal to noise ratio (S/N) of the TMT reporter ions peak in the MS/MS spectrum. The S/N signal of multiple distinct peptides from each protein was summed to report the protein signal.

The intra-run variation for each sample in the mix was normalized with the Proteome Discoverer software to get the total protein signal of each sample the same as the highest one in the run. The inter-run variation was normalized based on the identical control samples in each run, performed in R environment (Supplementary File 1).

Then, low-quality proteins with identification Score Sequest HT <5 were removed from the data set.

### Adjustment for blood protein contamination

To reduce the influence of the blood protein contamination on the AT proteome, we retrieved information from the UniProt database to set up a blood protein exclusion list (Supplementary Table 1) with known abundant blood-specific proteins^20^, including all immunoglobulins. The final signal was the protein abundance in a blood protein-free AT sample.

Final signal *[protein x*, *sample i]* = normalized signal *[protein x*, *sample i]* * Σ normalized signal *[sample i]*/(Σnormalized signal *[sample i]* − Σ Blood protein normalized signal *[sample i]*)

This final signal (below referred to as ‘signal’) was used in data analysis.

### Western Blotting

The paired biopsies from abdominal and femoral subcutaneous adipose tissue from the same overweight/obese individuals (n = 8) were used to perform Western Blotting for periostin (POSTN) and annexin A2 (ANXA2), which appeared to be differentially expressed between abdominal and femoral adipose tissue using LC-MS methodology, to confirm the LC-MS results. Samples with equal amount of protein were run on a 12% SDS polyacrylamide gel (180 V, Criterion Cell; Bio-Rad, Hercules, CA), then transferred (90 min, 100 V, Criterion blotter; Bio-Rad) to 0.45-mm nitrocellulose membranes. Total protein was stained with Ponceau S followed by destaining. Membranes were blocked in 5% non-fat dry milk power (NFDM; Bio-Rad) in Tris-buffered saline containing 0.1% Tween 20 (TBST) for 1 h. Thereafter, the blots were incubated with the primary antibodies against Periostin (1:1,000 dilution, AbCam) and AnnexinA2 (1:1,000 dilution, Santa Cruz) in 5% NFDM-TBST overnight at 4 °C on a shaker. Then, the blots were washed three times for 10 min in TBST and incubated for 1 h with a 1:10,000 dilution of the horseradish peroxidase-conjugated secondary antibody (DAKO) in 5% NFDM-TBST. A CCD camera (XRS-system, Biorad) was used to detect immunoreactive bands using chemiluminescent substrate (SuperSignal CL; Pierce). The quantification was performed with the program Quantity One version 4.6.5 (Bio-Rad). Ponceau S was used to standardize for the amount of protein loaded.

### Univariate statistics

For statistical analyses, missing values in proteome data were imputed with the half of the lowest positive signal in the dataset. Thereafter, data were log2 transformed. Two-sided paired Student’s t-test was used to test AT depot-differences for fat cell size and each protein detected by the LC-MS approach. False discovery rate (FDR) q-value was calculated to adjust proteomics data for multiple testing. Proteins with a p-value < 0.05 were regarded as differentially expressed, and were selected for further biological annotation and analysis.

A heat-map was generated based on these proteins using their scaled data (mean-centered value divided by standard deviations per protein). The Euclidean method was used to compute distance, and the ward.D method was used to compute the hierarchical clusters of proteins.

The association between fat cell size and protein expression was analyzed by Spearman’s rank correlation for both abdominal and femoral subcutaneous AT.

The protein expression determined by Western blotting was analysed by one-sided non-parametric Wilcoxon signed-rank test to confirm the differences found with LC-MS.

### Multivariate analysis

Hotelling’s T^2^-test for two dependent samples is the multivariate extension of the two-group paired Student’s t-test^21,22^. The proteome profile data of 610 proteins Log2 transformed expression values were first downscaled to 7 principle components (the maximal degree of freedom for 8 paired samples) by multidimensional scaling, and then entered the Hotelling’s T^2^-test. A p-value < 0.05 was considered to be statistically significant. All statistical analyses were performed in R environment, version 3.4.2, with various packages (stats, gplots, limma, ICSNP).

## Results

Subjects’ characteristics are shown in Table 1. By design, the overweight/obese (BMI, 34.4 ± 1.6 kg/m^2^) subjects included in the present study had an impaired glucose metabolism. Fat cell size was smaller in abdominal as compared to femoral subcutaneous AT (58.0 ± 3.3 vs. 65.9 ± 2.3 μm, p = 0.011) (Table 1).

**Table 1:** Subject characteristics (*n* = 8).

	Baseline Mean ± SEM
Age (yrs)	52.5 ± 1.8
Weight (kg)	99.6 ± 6.0
BMI (kg/m^2^)	34.4 ± 1.6
Body fat (%)	43.5 ± 1.1
Trunk fat mass (kg)	20.4 ± 1.9
Leg fat mass (kg)	15.9 ± 1.0
Waist circumference (cm)	115.9 ± 6.2
Waist/hip ratio	1.00 ± 0.03
Fasting glucose (mmol/L)	5.8 ± 0.1
2-h glucose (mmol/L)	6.1 ± 0.8
Abdominal fat cell size (μm)	58.0 ± 3.3
Femoral fat cell size (μm)	65.9 ± 2.3

**Figure 1:**
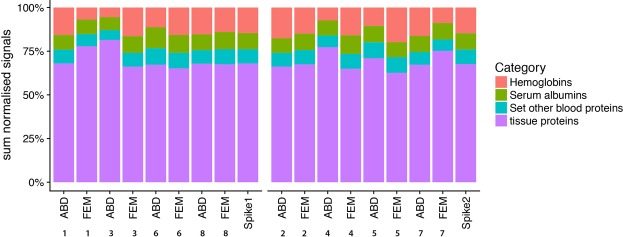
Percentage of signal belonging to tissue or different blood-specific protein groups. Each bar represents an adipose tissue biopsy, with the number representing a certain subject. ABD; abdominal, FEM; femoral. Spike refers to the control (pooled sample) in each run.

In total, 651 proteins with sufficient HT score were identified in the AT samples, and were subsequently quantified. Several of the identified proteins were blood-specific proteins (Supplementary Table 1) due to the presence of some blood in the whole-AT biopsies, despite thorough cleaning of the biopsies with sterile saline. We found that about one third (range 18–38%) of the total protein signals were attributable to blood-specific proteins, warranting a correction for blood contamination (Fig. 1). Data cleaning by removal of these blood-specific protein signals resulted in 610 identified AT proteins.

The total proteome with 7 principle components, which explained 92% of total variation of the 610 AT proteins, tended to be different between abdominal and femoral subcutaneous AT (p = 0.053) by multivariate Hotelling’s T^2^-test.

**Figure 2:**
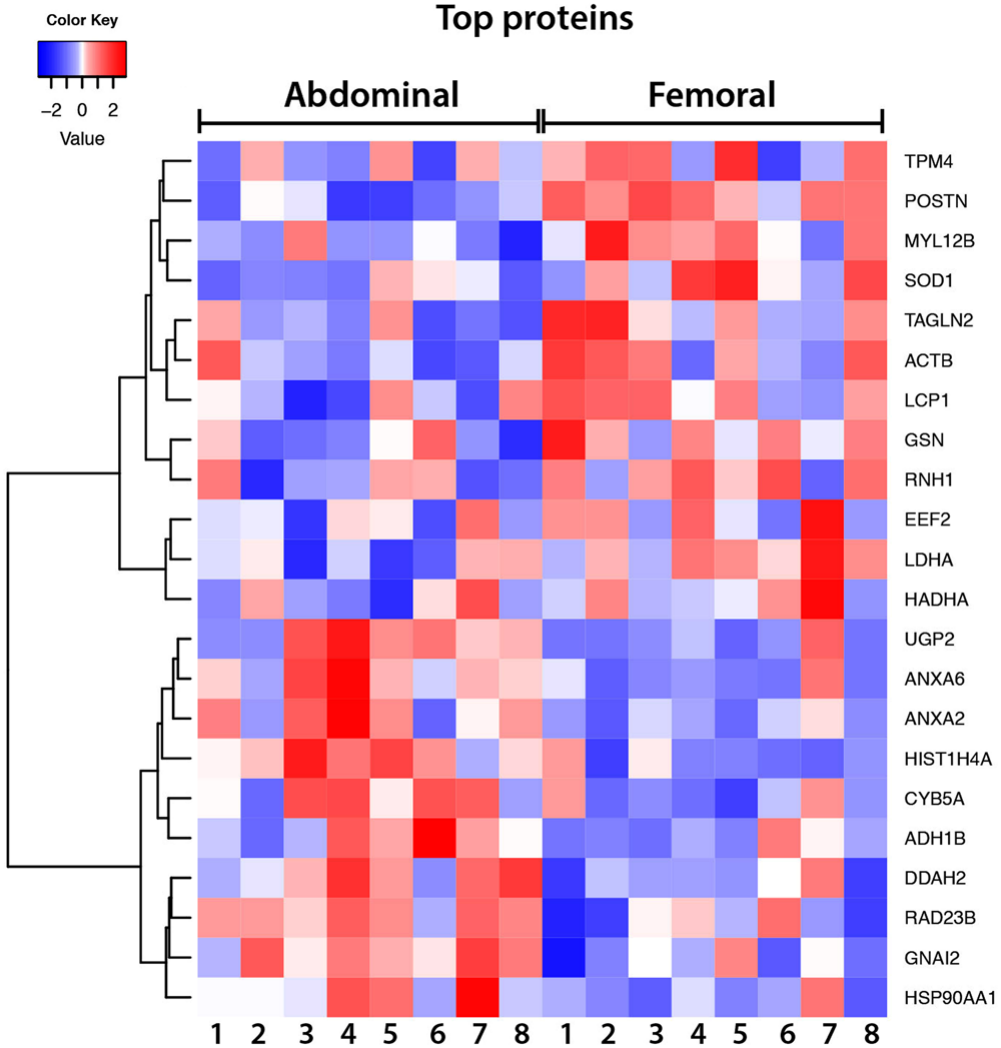
Heatmap of proteins that were differentially expressed between abdominal and femoral subcutaneous adipose tissue. Each cell represents one protein expression standardized score in one subject (organized by column, subjects are labelled by number). The color key is proportional to the protein expression standardized score.

Comparison of protein expression revealed that 22 of the 610 identified AT proteins tended to be differentially expressed between both AT depots (p < 0.05, but all q > 0.05, Table 2). A heat-map was constructed to visualize the pattern in protein expression between abdominal and femoral subcutaneous AT for these 22 proteins (Fig. 2). Individual differences in protein expression between these AT depots are shown in Supplementary Fig. 1.

**Table 2:** Proteins that tended to be differently expressed between abdominal and femoral adipose tissue (p-value < 0.05, q-value > 0.05).

Accession	Gene symbol	Full protein name	Fold-change ABD vs FEM	p-value
Q15063	POSTN	Periostin	0.80	<0.001
P13796	LCP1	Plastin-2	0.88	0.049
O14950	MYL12B	Myosin regulatory light chain 12B	0.89	0.047
P37802	TAGLN2	Transgelin-2	0.92	0.011
P00338	LDHA	L-lactate dehydrogenase A chain	0.92	0.010
P06396	GSN	Gelsolin	0.92	0.029
P00441	SOD1	Superoxide dismutase	0.93	0.028
P67936	TPM4	Tropomyosin alpha-4 chain	0.93	0.036
P40939	HADHA	Trifunctional enzyme subunit alpha, mitochondrial	0.93	0.019
P13489	RNH1	Ribonuclease inhibitor	0.93	0.021
P13639	EEF2	Elongation factor 2	0.94	0.010
P60709	ACTB	Actin, cytoplasmic 1	0.95	0.006
P07900	HSP90AA1	Heat shock protein HSP 90-alpha	1.05	0.003
O95865	DDAH2	N(G),N(G)-dimethylarginine dimethylaminohydrolase 2	1.08	0.047
P54727	RAD23B	UV excision repair protein RAD23 homolog B	1.09	0.037
P62805	HIST1H4A	Histone H4	1.11	0.004
P08133	ANXA6	Annexin A6	1.12	0.031
P07355	ANXA2	Annexin A2	1.13	0.043
P04899	GNAI2	Guanine nucleotide-binding protein G(i) subunit alpha-2	1.14	0.006
Q16851	UGP2	UTP–glucose-1-phosphate uridylyltransferase	1.19	0.032
P00325	ADH1B	Alcohol dehydrogenase 1B	1.22	0.012
P00167	CYB5A	Cytochrome b5	1.25	0.039

Several of the 22 identified proteins that were differentially expressed between femoral and abdominal AT were related to cellular structure, including the extracellular matrix (ECM). More specific, protein expression of periostin (POSTN), myosin regulatory light chain 12B (MYL12B), gelsolin (GSN), tropomyosin alpha-4 chain (TPM4), actin cytoplasmic 1 (ACTB), and actin-binding protein lymphocyte cytosolic protein 1 (LCP1) was higher in femoral as compared to abdominal AT. Moreover, some proteins related to energy metabolism were expressed at a higher level in femoral than abdominal AT, including mitochondrial hydroxyacyl-coenzyme A dehydrogenase (i.e. trifunctional enzyme subunit alpha, HADHA) and L-lactate dehydrogenase A chain (LDHA). In contrast, certain other proteins related to energy metabolism such as alcohol dehydrogense 1B (ADH1B), and cytochrome b5 (CYB5A), were expressed at a lower level in femoral versus abdominal AT. We also found a higher expression of protein synthesis complex component elongation factor 2 (EEF2) and redox superoxide dismutase (SOD1) in femoral AT. Furthermore, chaperone heat shock protein HSP 90-alpha (HSP90AA1), nitric oxide production related enzyme dimethylarginine dimethylaminohydrolase 2 (DDAH2), and glycogen synthesis enzyme UTP-glucose-1-phosphate uridylyltransferase (UGP2) were also expressed at a lower level in femoral as compared to abdominal subcutaneous AT.

Western blot analyses was performed, using the paired biopsies from abdominal and femoral subcutaneous adipose tissue from the same overweight/obese individuals (n = 8), to confirm the adipose tissue depot-differences in protein expression of POSTN and ANXA2 found using LC-MS. In line with the reported LC-MS data (Table 2), we found that the expression of POSTN was significantly lower in abdominal than femoral adipose tissue (FC = 0.75, p = 0.039) (Supplementary Fig. 2). Furthermore, protein expression of ANXA2 showed an expression pattern similar to that obtained using LC-MS, even at the individual level for most subjects (Supplementary Fig. 2), although this did not reach statistical significance (FC = 1.25, p = 0.27).

**Figure 3:**
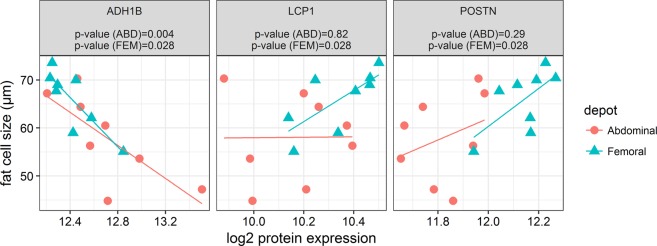
Correlation between proteins (AHDH1, LCP1, and POSTN) and fat cell size (μm) in abdominal and femoral subcutaneous adipose tissue.

Since the differentially expressed proteins seems to be related to processes of cellular structure and energy metabolism, we next investigated the association between fat cell size and protein expression of these differentially expressed proteins. Fat cell size in abdominal and femoral AT was negatively associated with ADH1B (p = 0.004 and p = 0.028, respectively), while fat cell size was positively correlated with LCP1 (p = 0.028) and POSTN (p = 0.028) in femoral AT (Fig. 3).

## Discussion

The aim of the present study was to investigate differences in the proteome of abdominal and femoral subcutaneous AT in overweight and obese women with impaired glucose metabolism, and to examine the associations between fat cell size and the AT proteome, since this may yield mechanistic insight into functional differences between these AT depots. For this purpose, quantitative LC-MS analysis of paired biopsies was performed to identify and quantify proteins. Here, we identified and quantified 610 proteins, and demonstrated that no major differences exist between the proteome of abdominal and femoral subcutaneous AT following an overnight fast. Nevertheless, 22 proteins seemed to be differentially expressed between abdominal and femoral AT, and fat cell size was significantly correlated with ADH1B, POSTN and LCP1.

We found that, using the PANTHER library of protein (sub)families^23^, the 22 differentially expressed proteins were related to ‘protein binding’ (8/22) and ‘catalytic activity’ (8/22) in the category ‘molecular function’. Moreover, in the category ‘biological processes’, the most prominent differences were related to ‘metabolic processes’ (13/22) and ‘cellular processes’ (11/22). In line with this global analysis, a more detailed examination of the expression of these proteins showed that differences in structural proteins may exist between abdominal and femoral AT, indicated by the lower abundance of the ECM protein POSTN and the cytoskeleton-related proteins MYL12B, TPM4, ACTB, GSN, TAGLN2, and LCP1 in abdominal as compared to femoral subcutaneous AT. Interestingly, POSTN was the only protein that showed consistent AT depot differences in all subjects, and also showed the most pronounced difference (~25% higher expression in femoral AT). According to mRNA expression data, POSTN is ubiquity expressed in most tissues, of which high expression levels were found in digestive organs/metabolic tissues, suggesting a relation with nutrient metabolism^24^. This protein functions as a cell adhesion component^25^, which may stimulate the maturation of ECM (by similarity, Uniport). POSTN is also highly expressed in collagen-rich connective tissue and has previously been associated with obesity^26^ and weight regain in females^27^. These findings suggest that POSTN may be involved in lipid storage in adipocytes, and repair and/or expansion of AT. POSTN is able to bind to integrins and can transduce external signals into cells via the focal adhesion kinase pathway^28^. Intracellularly, focal adhesions are attached to the actin filaments, which undergo a structural reorganization during the differentiation of adipocytes^29^. In this regard, both POSTN and ACTB may be involved in AT expandability or, in other words, the fat storage capacity of AT. Indeed, we found POSTN to be positively correlated with femoral but not abdominal fat cell size. In addition, the protein abundances of EEF2 and RNH1, a translation elongation factor and an inhibitor of mRNA turnover, were also lower in abdominal AT, which may be indicative of differences in protein synthesis accompanying tissue expansion. In agreement with these findings, fat cell size was significantly smaller in abdominal than femoral subcutaneous AT, as we have also reported previously^12^. The latter is in line with earlier studies showing larger femoral than abdominal adipocytes in obese men and women^30,31^. Adipocyte expansion by fat storage may lead to cell stress^32^. In this respect, a lower abundance of HSP90AA1 in femoral AT would be in line with a larger expansion capacity of femoral adipocytes, without being hampered by cell stress, although this remains to be elucidated. Altogether, the present proteome analysis may point towards key proteins involved in human AT remodelling.

Moreover, many of the proteins that had a lower abundance in abdominal subcutaneous AT are functionally related to actin filaments. GSN binds to the positive end of actin monomers and filaments, thereby preventing monomer exchange. TPM4 forms dimers, which interact with the actin filaments and controls the access of actin-associated proteins to the filaments^33,34^. MYL12B is a component of myosin II, which can form contractile structures in connection with actin filaments^35^. Its phosphorylation triggers formation of myosin II filaments but also actin polymerization. Both TAGLN2 and LCP1 are proteins which regulate actin filament polymerization^36,37^. Notably, both of these proteins may be involved in the formation of immunological synapses between leukocytes and target cells^38,39^. Taken together, the differences in protein expression levels of structural proteins may indicate that differences exist in tissue structure between upper- and lower-body AT depots in humans, and suggest a different tendency for the interaction of immune cells inside AT. This is further strengthened by the positive correlation between fat cell size and LCP1 in femoral AT.

Furthermore, we found that protein expression of cytoplasmic SOD1 was lower in abdominal than femoral AT. This protein plays a role in scavenging naturally occurring oxygen radicals. In accordance, RNH1, previously found to be implicated in protection against oxidative stress^40^, was also expressed at a lower level in abdominal subcutaneous AT. Together, these findings suggest that these subcutaneous AT depots may differ in the level of oxidative stress. Moreover, the lower abundance in HADHA and LDHA proteins in abdominal versus femoral AT further suggests differences in metabolic activity between these AT depots, since these proteins are involved in mitochondrial beta-oxidation and anaerobic glycolysis, respectively. Furthermore, other metabolic enzymes (ADH1B and CYB5A) appeared to be higher expressed in abdominal AT, implying pathway-specific differences in the expression of proteins involved in metabolic activity between both fat depots. Interestingly, protein expression of ADH1B, of which gene expression is highest in AT as compared to other tissues^24^, was negatively correlated with abdominal and femoral fat cell size. Moreover, ADH1B has also been implicated in body weight regulation^41^, and was negatively correlated with waist circumference, BMI and fasting plasma insulin^42^. Taken together, it is tempting to suggest that ADH1B may be involved in AT expandability.

The strength of the present study is that we, for the first time to our knowledge, compared the proteome of human abdominal and femoral subcutaneous AT. Moreover, the paired comparisons that were made between both subcutaneous AT depots for each individual further strengthen this study. Thus far, very few proteomic analyses of human whole-AT have been performed. Despite thorough cleaning, the AT biopsies still contained residual blood, which resulted in the identification of several blood proteins that accounted for ~20–30% of the total AT protein signal. Blood contamination is not surprising, since AT is a relative highly vascularized tissue. Unavoidable blood contamination might be one of the barriers to obtain a real ‘AT proteome’, but we adjusted for blood proteins in the present analyses. Furthermore, Western Blot analyses confirmed the adipose tissue depot-differences in protein expression of POSTN and, to a lesser extent, ANXA2 that were found using LC-MS. Noteworthy, the data obtained using Western blotting showed more variation between samples as compared to the LC-MS results. The latter suggests that the LC-MS methodology that we have employed in the present study is more sensitive to detect adipose tissue depot-differences in protein expression, which may partly be due to the adjustment for blood protein contamination.

A limitation of the present study is the relatively small number of participants. Furthermore, our proteomic analysis covered only a small amount of proteins of the AT proteome, based on human adipose tissue transcriptome data (https://www.proteinatlas.org/humanproteome/adipose). Thus, our findings may reflect only part of the AT depot proteome differences. Furthermore, AT depot differences in protein abundance did not yield significant findings following adjustment for multiple testing in the present study, which might be due to limited power and/or subtle differences in protein expression between these AT depots. Therefore, the differences reported here should be confirmed in future studies, also taking into account that certain differences in protein expression between adipose tissue depots may be either more or less pronounced depending on the study population. Here, we studied adipose tissues from metabolically compromised individuals, because we expected this to increase potential differences between the depots. However, it might well be that a disturbed glucose metabolism masks any depot differences in protein expression that might exist among individuals with a different metabolic phenotype (e.g. healthy subjects). Finally, AT biopsies were collected following an overnight fast. Therefore, it may well be that under challenged conditions such as after physical exercise, prolonged fasting or a dietary intervention, proteome differences are more outspoken. Noteworthy, protein abundance may not always be a valid surrogate for protein activity and, as such, our observations warrant follow-up research.

In conclusion, comparison of human abdominal and femoral subcutaneous AT using non-targeted, quantitative proteomics revealed slight but specific differences in protein expression between these AT depots in overweight/obese women, and indicated that fat cell size was significantly correlated with ADH1B, POSTN and LCP1. These differences in protein expression may reflect depot-differences in adipocyte morphology, adipocyte expandability, immune cell interaction and energy metabolism. Importantly, it cannot be excluded that differences in protein activity, particularly under challenged conditions, contribute to divergent functioning of these AT depots.

## Supplementary information

Supplementary file
