# Supplementary material for: A comparison between the abdominal and femoral adipose tissue proteome of overweight and obese women
Source: Sci Rep. 2019 Mar 12;9:4202. doi: 10.1038/s41598-019-40992-x (PMC6414508; doi:10.1038/s41598-019-40992-x)
Supplement: Supplementary file 1 — Supplementary file [file 41598_2019_40992_MOESM1_ESM.docx]

**A comparison between the abdominal and femoral adipose tissue proteome of overweight and obese women**

First author: Vogel

M.A.A. Vogel^1^, P. Wang^1,2^, F.G. Bouwman^1^, N. Hoebers^1^, E.E. Blaak^1^, J. Renes^1^, E.C. Mariman^1^ and G.H. Goossens^1^

*^1^Department of Human Biology, NUTRIM School of Nutrition and Translational Research in Metabolism, Maastricht University Medical Center^+^, Maastricht, The Netherlands*

*^2^ Department of Clinical Genetics, Maastricht University Medical Center^+^, Maastricht, The Netherlands*

**Correspondence:**Dr. Gijs H. Goossens, PhD

Department of Human Biology, NUTRIM School of Nutrition and Translational Research in Metabolism, Maastricht University Medical Centre^+^, PO Box 616, 6200 MD, Maastricht, The Netherlands, Fax: +31 43 3670976, Phone: +31 43 3881314, Email: [G.Goossens@maastrichtuniversity.nl](mailto:G.Goossens@maastrichtuniversity.nl)

**Supplementary Table 1:** Identified blood proteins in the AT biopsies.

| **Accession** | **Gene symbol** | **Full protein name** |
| --- | --- | --- |
| P02768 | ALB | Serum albumin |
| P69905 | HBA1 | Hemoglobin subunit alpha |
| P68871 | HBB | Hemoglobin subunit beta |
| P02549 | SPTA1 | Spectrin alpha chain, erythrocytic 1 |
| P02787 | TF | Serotransferrin |
| P01023 | A2M | Alpha-2-macroglobulin |
| P01009 | SERPINA1 | Alpha-1-antitrypsin |
| P0DOX5 |  | Immunoglobulin gamma-1 heavy chain |
| P00738 | HP | Haptoglobin |
| P02042 | HBD | Hemoglobin subunit delta |
| P00450 | CP | Ceruloplasmin |
| P01871 | IGHM | Immunoglobulin heavy constant mu |
| P69891 | HBG1 | Hemoglobin subunit gamma-1 |
| P01876 | IGHA1 | Immunoglobulin heavy constant alpha 1 |
| P01860 | IGHG3 | Immunoglobulin heavy constant gamma 3 |
| P0DOY2 | IGLC2 | Immunoglobulin lambda constant 2 |
| P02763 | ORM1 | Alpha-1-acid glycoprotein 1 |
| P0DOX7 |  | Immunoglobulin kappa light chain |
| P01834 | IGKC | Immunoglobulin kappa constant |
| P01859 | IGHG2 | Immunoglobulin heavy constant gamma 2 |
| P00747 | PLG | Plasminogen |
| P19652 | ORM2 | Alpha-1-acid glycoprotein 2 |
| P02766 | TTR | Transthyretin |
| P01861 | IGHG4 | Immunoglobulin heavy constant gamma 4 |
| P0DOX8 |  | Immunoglobulin lambda-1 light chain |
| P01780 | IGHV3-7 | Immunoglobulin heavy variable 3-7 |
| A0A0C4DH34 | IGHV4-28 | Immunoglobulin heavy variable 4-28 |
| P01768 | IGHV3-30 | Immunoglobulin heavy variable 3-30 |
| P09105 | HBQ1 | Hemoglobin subunit theta-1 |
| A0A0C4DH25 | IGKV3D-20 | Immunoglobulin kappa variable 3D-20 |
| P01619 | IGKV3-20 | Immunoglobulin kappa variable 3-20 |
| A0A0C4DH68 | IGKV2-24 | Immunoglobulin kappa variable 2-24 |
| P01764 | IGHV3-23 | Immunoglobulin heavy variable 3-23 |
| P69892 | HBG2 | Hemoglobin subunit gamma-2 |
| P01824 | IGHV4-39 | Immunoglobulin heavy variable 4-39 |
| P01591 | JCHAIN | Immunoglobulin J chain |
| A0A0B4J1X5 | IGHV3-74 | Immunoglobulin heavy variable 3-74 |
| P01877 | IGHA2 | Immunoglobulin heavy constant alpha 2 |
| P04220 |  | Ig MU heavy chain disease protein |
| P02100 | HBE1 | Hemoglobin subunit epsilon |
| P80748 | IGLV3-21 | Immunoglobulin lambda variable 3-21 |

1

2

3

4

5

6

7

8


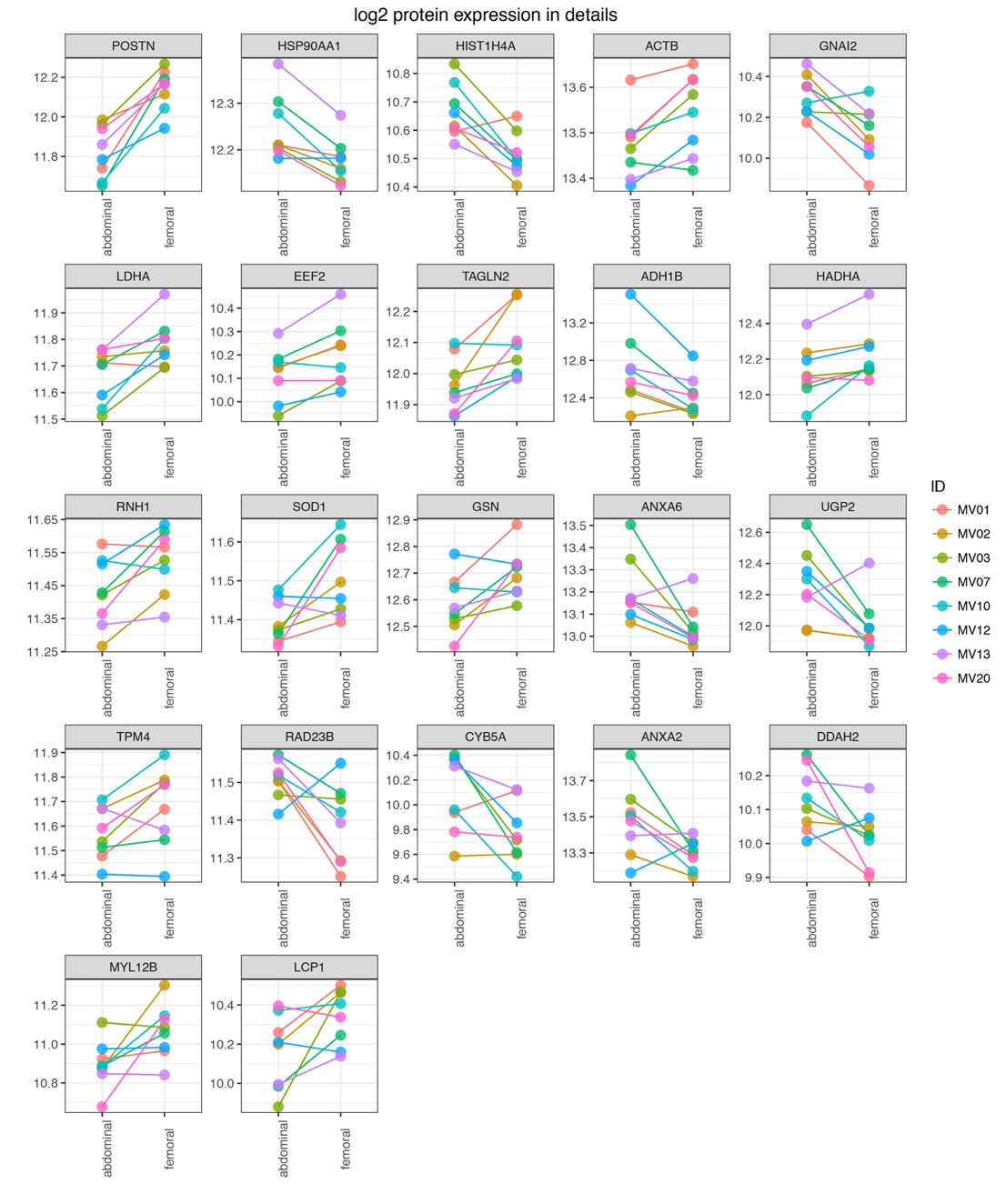


**Supplementary Figure 1.** Top 22 identified proteins that were differentially expressed between abdominal and femoral subcutaneous adipose tissue (p < 0.05). Expression data (n=8) are illustrated for each individual (n=8) separately.

**Supplementary Figure 2.** Protein expression of periostin (POSTN) and Annexin A2 (ANXA2) using LC-MS and Western Blotting (WB) in paired samples from abdominal and femoral subcutaneous adipose tissue (n=8), indicating protein expression levels for each individual. Western Blotting demonstrated that POSTN expression was significantly lower in abdominal as compared to femoral adipose tissue (FC=0.75, p=0.039), which confirms the results obtained using LC-MS (P<0.05). Protein expression of ANXA2 using Western Blotting also revealed an expression pattern similar to that obtained with LC-MS (P<0.05), although this did not reach statistical significance (FC=1.25, p=0.27).

**Supplementary File 1**

**Inter-run data normalization:** The inter-run variation was normalized per protein based on the identical control samples spiked in each run, performed in R environment v3.4.

- If signals existed in multiple runs and also in control samples: correction is based on the signal of this specific protein.

Normalized signal *[protein x, sample i, run j*]= signal [*protein x, sample i, run j*]/

(signal [*protein x, control, run j*]/median (signal [*protein x, controls, run 1:m*]))

- If signals existed in multiple batches but not in (one of the) control samples: correction is based on total signal of the sample.

Normalized signal [protein x, sample i, run j]= signal [protein x, sample i, run j]/

(Σ signal [control, run j]/median (Σ signal [controls, run 1:m]))

- If no signal existed in the sample, it is left as empty.

**R code**

# normalized signal=raw signal/batch correction factor

my.normalization <- function(x, y) {

obj<-try(x/y, silent=TRUE)

if (is(obj, "try-error")) return(NA) else return(obj)

}

# dat.raw is a dataframe of all raw signals of all samples including control and real sample

# with columns of "accession" , "sample", "signal", "Batch", "sample.type", “Quality”

library(reshape2)

dat<-dcast(dat.raw, Batch+sample.type+sample~accession, value.var="signal",fun.aggregate = sum)

dat[dat==0]<-NA

dat$Batch<-as.factor(dat$Batch)

dat.control<-dat[dat$sample.type=="control",]

sums<-as.data.frame(rowSums(dat.control[,c(4:ncol(dat.control))],na.rm = TRUE))

names(sums)<-"sum"

if(nrow(sums)>1 & length(levels(dat$Batch))>1){

# exist multiple batch and control samples

dat.control<-cbind(dat.control,sums)

summedian<-median(dat.control$sum, na.rm=TRUE)

dat.control$sumcoefficient<-dat.control$sum/summedian # overal batch ratio coefficient

for (i in 4:ncol(dat)){

# normalize protein one by one

protmedian<-median(dat.control[,i], na.rm=TRUE)

for (b in 1: length(dat.control$Batch)){

if(!is.na(dat.control[dat.control$Batch==levels(dat.control$Batch)[b],i])){

# protein is present in this batch control

# batch correction factor for this protein

c=dat.control[dat.control$Batch==levels(dat.control$Batch)[b],i]/protmedian

} else {

# protein is absent in the batch control

# batch correction factor for this protein

c=dat.control$sumcoefficient[dat.control$Batch==levels(dat.control$Batch)[b]]

}

dat[dat$Batch==levels(dat.control$Batch)[b],i]<- my.normalization(dat[dat$Batch==levels(dat.control$Batch)[b],i],c)

}

}

dat<-dat[order(dat$sample.type),]

dat.melt<-melt(dat[,c(3:ncol(dat))], id.vars ="sample" )

dat.melt$proteinID<-interaction (dat.melt$variable,dat.melt$sample)

# signals after normalization

dat.norm<-dat.raw

dat.norm$proteinID<-interaction (dat.norm$accession,dat.norm$sample)

dat.norm$signal<-dat.melt$value[match(dat.norm$proteinID,dat.melt$proteinID)]

}
